# High-throughput image-based monitoring of cell aggregation and microspheroid formation

**DOI:** 10.1371/journal.pone.0199092

**Published:** 2018-06-28

**Authors:** Thomas Deckers, Toon Lambrechts, Stefano Viazzi, Gabriella Nilsson Hall, Ioannis Papantoniou, Veerle Bloemen, Jean-Marie Aerts

**Affiliations:** 1 M3-BIORES, KU Leuven, Leuven, Belgium; 2 Biomedical-Health Engineering, KU Leuven Campus Group T, Leuven, Belgium; 3 Prometheus, Division of Skeletal Tissue Engineering Leuven, KU Leuven, Leuven, Belgium; 4 Skeletal Biology and Engineering Research Center, KU Leuven, Leuven, Belgium; University of Bergen, NORWAY

## Abstract

Studies on monolayer cultures and whole-animal models for the prediction of the response of native human tissue are associated with limitations. Therefore, more and more laboratories are tending towards multicellular spheroids grown *in vitro* as a model of native tissues. In addition, they are increasingly used in a wide range of biofabrication methodologies. These 3D microspheroids are generated through a self-assembly process that is still poorly characterised, called cellular aggregation. Here, a system is proposed for the automated, non-invasive and high throughput monitoring of the morphological changes during cell aggregation. Microwell patterned inserts were used for spheroid formation while an automated microscope with 4x bright-field objective captured the morphological changes during this process. Subsequently, the acquired time-lapse images were automatically segmented and several morphological features such as minor axis length, major axis length, roundness, area, perimeter and circularity were extracted for each spheroid. The method was quantitatively validated with respect to manual segmentation on four sets of ± 60 spheroids. The average sensitivities and precisions of the proposed segmentation method ranged from 96.67–97.84% and 96.77–97.73%, respectively. In addition, the different morphological features were validated, obtaining average relative errors between 0.78–4.50%. On average, a spheroid was processed 73 times faster than a human operator. As opposed to existing algorithms, our methodology was not only able to automatically monitor compact spheroids but also the aggregation process of individual spheroids, and this in an accurate and high-throughput manner. In total, the aggregation behaviour of more than 700 individual spheroids was monitored over a duration of 16 hours with a time interval of 5 minutes, and this could be increased up to 48,000 for the described culture format. In conclusion, the proposed system has the potential to be used for unravelling the mechanisms involved in spheroid formation and monitoring their formation during large-scale manufacturing protocols.

## Introduction

Traditional monolayer cultures are increasingly recognized as insufficient to exhibit crucial physiological processes present in native tissues [1][2]. As an alternative, researchers often rely on animal models [3,4]. However, these *in vivo* experiments are accompanied by ethical and economic concerns. In addition, their predictive capabilities are limited due to differences in response between species [5]. As a result, three-dimensional (3D) cell cultures, and in particular multicellular spheroids [6], are currently used in a broad range of biomedical applications [7,8]. *In vitro* spheroids mimic the structural and functional characteristics of native tissues more closely, thereby bridging the gap between monolayer cultures and *in vivo* animal models [9]. They have been demonstrated to serve as reliable *in vitro* models for toxicity screening [10], radiation therapy [11], drug discovery [12], dosage studies [13], cancer research and tissue morphogenesis [3]. Moreover, in the field of tissue engineering, spheroids are extensively investigated for use in cell-based therapies (e.g. the stimulation of cartilage defect repair *in vivo* [14]) or as building blocks for *in vitro* fabrication of functional macro-tissues and organs [15]. At the basis of all these applications lies the cell aggregation process that leads to the formation of these spheroids [16]. This process can be described by Steinberg’s Differential Adhesion Hypothesis (DAH), which states that self-assembly is driven by surface tension, intercellular adhesion and cells’ liquid-like properties. When cells are brought into a non-adhesive environment, they attach to each other and organise themselves into a spheroid in order to minimise their surface free energy [17][18][19][20].

In general, the process behind cellular aggregation is still poorly understood and characterised. Studying the morphological changes during spheroid formation could provide an important source of information and can lead to new insights in the self-assembly of spheroids [21][22]. Moreover, it is also important to consider the morphology of the spheroids for their use in a wide range of biofabrication methodologies. In tissue engineering, for example, it is essential to have uniformly sized spheroids [6] with a maximum restriction on the size in order to make them dispensable through a bio-printer nozzle [15]. In addition, it is observed that the differentiation potential, rate of cell death and proliferation of embryonic stem cells is affected by spheroid size [23][24][25][26][27][28][29][30].

Optical microscopy is commonly used to capture the morphological features of compact spheroids [9], but also during cell aggregation [21]. Methods to quantify the morphology include the use of a microscope and an ocular micrometre [14,31], or by manually drawing the border of the spheroid and extracting the features using image analysis [21][32]. Those processes are both very time-consuming and labour-intensive. Therefore, the need for automated, high-throughput monitoring systems is growing in order to facilitate the analysis of large-scale, spheroid-based assays [33].

Over the last years, several systems (i.e. integration of the microscopic set-up, culture format and image analysis software) have been described in the literature for automatically extracting morphological features of readily formed spheroids. Regarding the microscopic set-up, the use of fluorescence microscopy has been proposed for spheroid analysis [33]. Although it can play a valuable role in scientific research, the fluorescent staining can disrupt the natural cell environment and limits the spheroids’ clinical applicability. In Piccinini *et al*. [34], a standard widefield microscope (i.e. not equipped with an automated scanning stage) was used for the acquisition of bright-field images containing a single conic well. This imposes a lot of manual work, unfavourable for high-throughput analysis. For monitoring purposes, individual spheroids are often cultured in separate wells of a microtiter plate (e.g. a 24 or 96 well plate with one centrally located spheroid in each well) [12][34][35][36]. In these cases, the scale of production is restricted by the culture format, interfering with the need for high-throughput monitoring. A higher throughput can be achieved in e.g. spinner flask cultures [31] and low-adherent, flat-bottomed plates [33][37][38][39]. However, these formats give rise to non-uniformly sized spheroids due to a lack of size control [16][40][41]. In addition, the spheroids are not isolated in separate wells, imposing additional challenges for spheroid segmentation and tracking. The software tool ImageJ [42] has frequently been used for the analysis of spheroids [31][43][44][45]. It is an image analysis platform applicable for a wide range of cell-based assays. However, using ImageJ often results in a manual or semi-automated approach. In contrast to this, Vinci *et al*. [35] described a system (i.e. the CeligoTM cytometer) able to automatically monitor the morphological changes of compacted spheroids.

The proposed systems to date are still limited in their throughput focusing on monitoring readily formed spheroids and are not developed for continuous monitoring of the single cell aggregation process present at the onset of spheroid formation. Therefore, our goal is to develop an automated, non-invasive system for high-throughput monitoring of the morphological changes that occur during this initial state of single cell aggregation to spheroids. The system should be applicable to both biomedical research and large-scale bio-manufacturing of spheroids.

## Materials and methods

### Live cell monitoring system (LiMSy)

**Fig 1:**
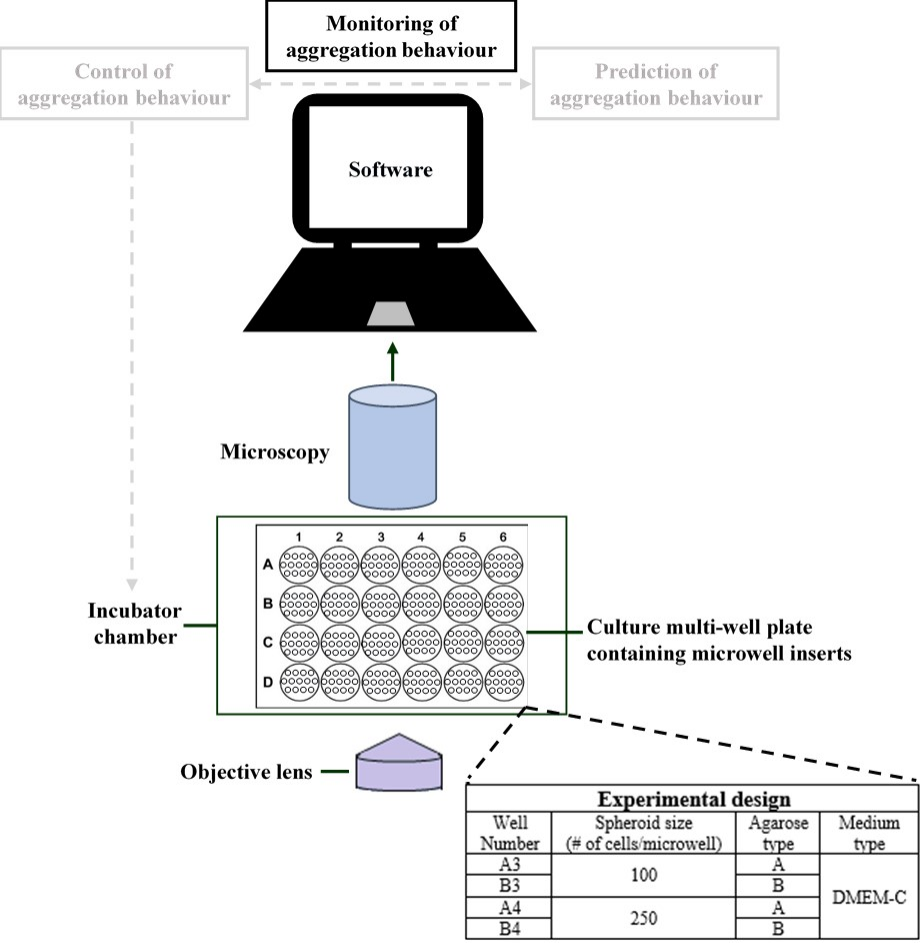
Experimental set-up for high-throughput monitoring of spheroid formation. The system is composed of three major components: (i) a microscopic set-up equipped with a motorized stage and an incubator chamber. (ii) a well plate containing agarose microwell inserts for spheroid culturing. (iii) a computer with image acquisition and analysis software. In the bottom-right corner, an overview of the culture conditions applied in this study is shown. For each condition, the corresponding well number of the 24 well plate is indicated.

In this study, we developed a system (Fig 1) able to continuously monitor the single cell aggregation process in an automated and high-throughput manner. An automated stage scanning microscope, consisting of a bright-field objective (Olympus IX53) with 4X magnification and a SC30 Colour Camera (Olympus), was used for image acquisition. Microspheroids were produced in microwell patterned inserts compatible with high-quality imaging and continuously monitored in a well plate incubator (OKOlab Top Stage Incubator with insert H301-EC-24MW). The culture conditions were actively controlled using an OKO-Air-Pump-BL, OKOlab 2GF-Mixer, and OKOlab T unit, all operated via an OKO Touch. For spheroid culturing, a temperature of 37°C and a CO_2_ concentration of 5% were applied in a humidified incubator. The acquired images were further processed using image analysis software.

### Fabrication of micro-patterned inserts

Agarose (3% w/v) was poured onto a master mold of polydimethylsiloxane (Dow Corning Sylgard 184 elastomer, MAVOM Chemical Solutions) to fabricate microwell imprinted disks, containing cylindrical, flat-bottomed microwells with a diameter of 200 micrometre and an inter-microwell space of 100 micrometre. A sterile puncher with a diameter of 15 mm was used to punch out inserts for a 24 well culture plate, containing approximately 2,000 microwells per insert. The inserts were placed in wells of a 24 well plate, 1 ml of phosphate buffered saline (PBS; Lonza) was added to each well and the well plate was sterilized under ultra-violet light for approximately 30 minutes. A more detailed description of the protocol is found in Leijten *et* al. [46].

### Cell culture and spheroid formation

Human periosteum-derived stem cells (hPDCs) were obtained from periosteal biopsies as described by Roberts *et al*. [47], with informed consent from the patient and approved by the Ethics Committee for Human Medical Research (KU Leuven). Furthermore, all experiments and methods were performed in accordance with the relevant guidelines and regulations. The cells were expanded in T175 tissue culture flasks (Greiner Bio-One) with a seeding density of 5 700 cells/cm^2^ and sub-cultured at ± 80% confluency. At 80% confluence, the cells were harvested by trypsinisation for 10 min with TrypLE^TM^ Express (Invitrogen). The medium used for expansion is a standard culture medium consisting of high glucose GlutaMAX^TM^ Dulbecco’s modified Eagle’s medium (DMEM; Life Technologies, Merelbeke, Belgium) supplemented with 10% irradiated fetal bovine serum (FBS; HyClone), 1% sodium pyruvate (Invitrogen) and 1% antibiotic–antimycotic (100 units/ml penicillin, 100 mg/ml streptomycin, and 0.25 mg/ml amphotericin B; Invitrogen). After passage 7, the cells were stored at -196°C in liquid nitrogen. Prior to the initialisation of the spheroid experiments, the cells were thawed, expanded for one passage and harvested with TrypLE. Subsequently, a hemocytometer (Neubauer) was used to manually count the cells, where live cells were distinguished from dead cells by adding trypan blue (Klinipath). During cell culture, the conditions of the incubator were actively controlled at 37°C, 95% relative humidity and 5% CO_2_.

In this study, the aggregation process was monitored for two different spheroid sizes (i.e. 100 and 250 cells/microwell) in combination with two different types of agarose: Eurogentec (Type A) and Invitrogen 15510–019 (Type B). An overview of the experimental design is shown in Fig 1. These process conditions served as a proof of concept to explore differences in aggregation kinetics and spheroid morphology related to the variable micro-environment of the cells. Spheroids composed of 100 or 250 cells were produced by adding 1 ml of cell suspension (suspended in DMEM-C), respectively containing 200,000 or 500,000 cells, drop-wise to the corresponding wells.

### Image acquisition

The cellSens Dimension (Olympus) software was used for image acquisition. Time-lapse datasets of the cell aggregation process were acquired at three random locations in each well (Fig 1), applying a time interval of 5 minutes. The experiment lasted for 16 hours in total, as previous unpublished data showed that cell aggregation reached a steady-state condition within this time frame. A focus map was used in combination with a resolution of 1532x2048 pixels to obtain sufficient image quality. Each dataset consisted of 193 images (i.e. every 5 minutes during 16 hours).

### Image analysis

A software tool was developed to automatically process the acquired images. The implementation of the algorithm was carried out in Matlab 2016b (MathWorks, MA, USA) using the Image Processing Toolbox^TM^. It was composed of two major parts: (I) The multicellular spheroids were automatically separated from the background using microwell detection and image segmentation techniques. Subsequently, the segmented spheroid masks were used to extract the features of interest. (II) The spheroids were tracked over time to obtain the dynamic responses of the individual spheroids.

**Fig 2:**
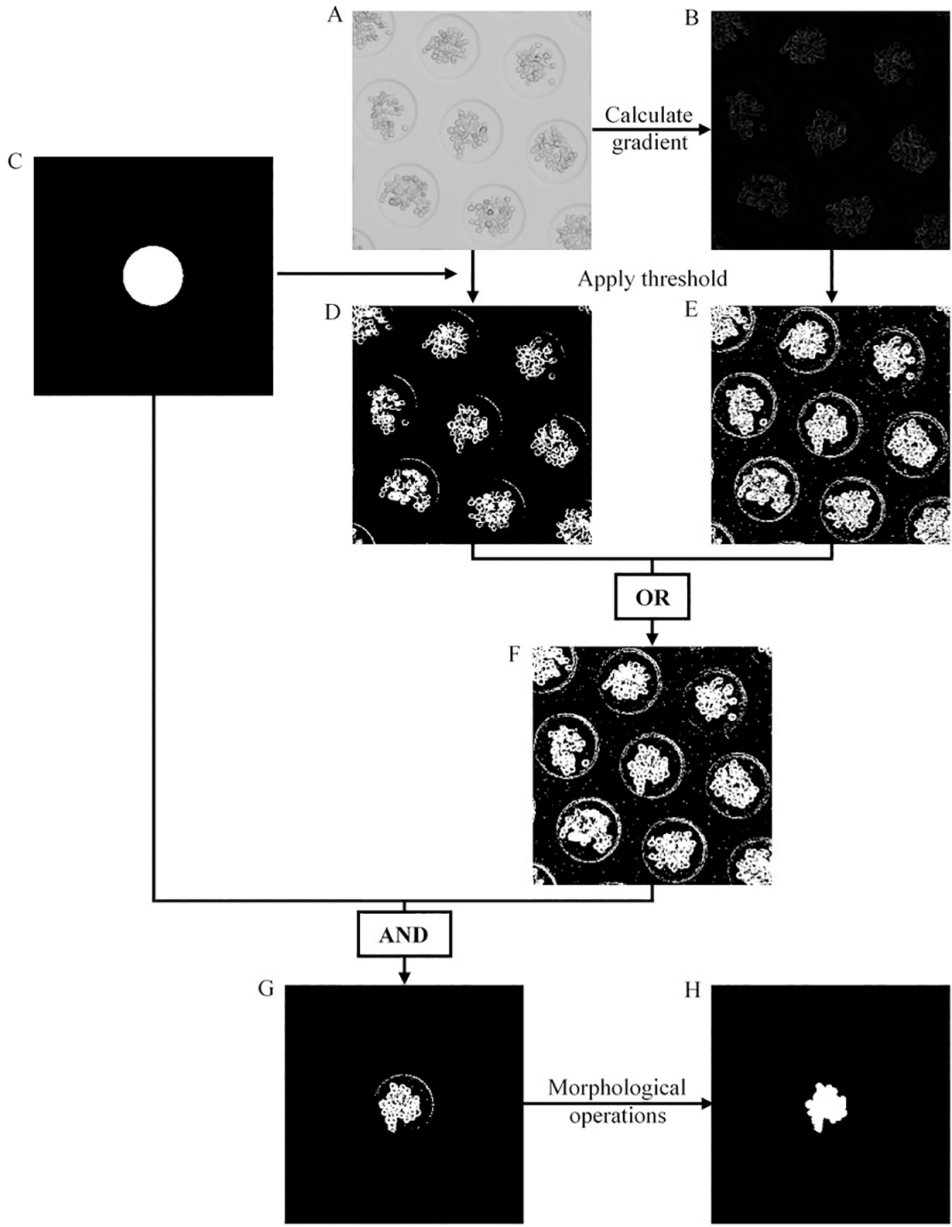
General overview of the proposed segmentation approach. (A) Raw image. (B) Gradient image computed from the raw image using Roberts gradient operator [50]. The gradient output was not normalised. (C) Circular mask of detected microwell. For further operations, the radius of the circular mask was set to the inner radius of the microwell (i.e. 75 pixels using the described microscopic set-up and microwells with a diameter of 200 *μm*). (D) The microwell mask was used to extract the intensity values of the corresponding spheroid and its surroundings (i.e. the background inside the microwell). Based on these intensity values, an intensity threshold was automatically computed using Otsu’s method [51]. This adaptive threshold was used to segment the raw image. (E) Evaluating the noise of the background, the gradient image was segmented with a fixed threshold of 0.020. (F) Both segmented images (D, E) were combined using an OR-operator. (G) The spheroid of interest (the one corresponding to the circular mask) was extracted using an AND-operator. (H) The final spheroid mask was obtained by applying the following morphological operations: (i) The largest object was retained together with all the objects that have an area smaller or equal to 25 pixels. (ii) Unconnected pixels were bridged and the resulting mask was diagonally filled to eliminate the 8-connectivity of the background. (iii) Holes smaller or equal to 150 pixels were filled, objects were morphologically eroded with a disk of radius 2 pixels, the largest object was retained and then morphologically dilated with a disk of radius 2 pixels. (iv) Prior to filling up the remaining holes, the mask was bridged and diagonally filled for a second time.

The detection of the microwells was achieved with the circle-searching function ‘imfindcircles’ based on the two-stage circular Hough transform [48]^,^[49], implemented in the Image Processing Toolbox^TM^ of Matlab. The raw image was converted to a gradient image using Roberts gradient operator [50]. The gradient image was thresholded with a threshold of 0.015 and the resulting image was used for microwell detection. Prior to execution, three parameters were specified for circle detection: the radius range, the sensitivity factor and the edge threshold, respectively set at 80–86 pixels, 0.975 and 0.030. The radius range, specified to detect the outer radius of the microwells, depended on the microwell diameter and the specifications of the microscope. The output matrices contained the centre coordinates (i.e. x- and y-coordinate) and radii of the detected circles. The removal of false positives was based on two criteria: the relative ‘circle strength’ (i.e. number of votes the circle received in the Hough accumulator, a metric assigned to each detected circle) and the fixed inter-microwell spacing. Furthermore, only the microwells that were completely inside the field of view were considered. In the next step, the spheroids were automatically segmented according to Fig 2. Morphological features such as the minor axis length, major axis length, roundness, area, perimeter and circularity were extracted from the final spheroid mask by aid of the ‘regionprops’ function in Matlab. In S1 Table, an overview of these features is listed along with their description. For each spheroid, the frame index and centre coordinates of its corresponding microwell were stored. This information was necessary to enable microwell tracking (see next paragraph). It was also possible to extract additional features, listed in S1 Table. Spheroid segmentation (Fig 2) and subsequent feature extraction were repeated for all the detected microwells. The features of all the spheroids in the current frame were stored in a matrix. The entire procedure is repeated for all the images in the dataset and the obtained matrices were stored in a time-stamped cell array.

Subsequently, the microwells (and therefore the corresponding spheroids) were automatically tracked over time. The tracking approach was based on the assumption that the shift of the microwells’ centre between subsequent frames was not larger than the microwell radius (plus an additional constant due to a fixed inter-microwell spacing). The dynamic response of each spheroid was stored in a separate matrix. A spheroid entering the field of view was assigned to a new matrix, regardless of the fact that it was in the field of view at a previous time point.

### Quantitative assessment

Manual segmentation is the gold standard to quantitatively validate a segmentation algorithm [52]. The open-source function ‘imfreehand’ (Matlab) allowed the operator to hand-draw the boundary of spheroids. From the resulting spheroid masks, the required features and pixel locations were extracted. The same operations were performed on the automatically generated masks. Subsequently, the following measures were computed to validate the proposed method: (i) the relative error on the automatically extracted morphological features (ii) sensitivity and precision of the proposed segmentation method, which are both described hereafter. All calculations were with respect to the manual segmentation of the same operator.

The captured spheroids range from loosely connected cells at the initial stage (i.e. first frame) towards packed spheroids at the final stage (i.e. last frame) of the experiment. In addition, the two different spheroid sizes were considered. Therefore, the validation was performed on four sets: Initial (Set 1) and Final (Set 2) small-sized spheroids, Initial (Set 3) and Final (Set 4) large-sized spheroids. The last defined dataset of the small and large-sized spheroids in the image acquisition software was used for validation (i.e. well B3 and B4 at location three, Fig 1). For each set, containing approximately 60 spheroids, the results were presented as mean ± standard deviation (SD).

### Relative error

In order to assess the reliability of the morphological feature estimation, the relative error was calculated for the different features of each spheroid according to Eq (1):

$$Relative\;error\;(\%)=\left|\frac{MS-AS}{MS}\right|*100$$ (1)

Where MS and AS were the values (units in S1 Table) obtained for each feature by respectively performing manual and automatic segmentation.

### Sensitivity and precision

Starting from the pixel coordinates of the segmented spheroids (for both manual and automatic segmentation), the sensitivity (true positive rate, TPR) and the precision (positive predictive value, PPV) were calculated for each spheroid according to Eqs (2) and (3):

$$TPR=\frac{TP}{TP+FN}$$ (2)

$$PPV=\frac{TP}{TP+FP}$$ (3)

True positive (TP) was the number of pixels correctly assigned to the spheroid by the algorithm, false positive (FP) was the number of pixels wrongly identified as being part of the spheroid and false negative (FN) was the number of spheroid pixels that have been missed with automatic segmentation.

The automated segmentation approach was qualitatively assessed on a cropped section of the validation images. Semi-transparent, red-coloured spheroid masks, segmented by the algorithm, were overlaid on their corresponding raw image. The abovementioned operations were executed in Matlab.

### Time consumption

The time required for manual segmentation of one spheroid was estimated over the four validation sets. The average time of the algorithm for processing a single spheroid was obtained by dividing the computation time for the four validation sets by the total number of analysed spheroids. Then, the total time consumption for the validation sets and the entire data was computed by multiplying the time requirements for one spheroid with the total number of spheroids to be analysed.

### Feature processing on population and spheroid level

For each condition (i.e. four in total), the dynamic response of the spheroid population was obtained by averaging the individual responses of approximately 180 spheroids. This operation was performed for the different features (S1 Table). Additionally, a video object (5 frames per second, S1 File) was constructed to illustrate the off-line monitoring of the aggregation process of individual spheroids. Screenshots were taken after 6 and 12 hours.

## Results

### Establishment of a system for high-throughput monitoring of spheroid formation

The spheroids composed of 100 and 250 cells were successfully cultured on the microwell format, as previously reported by Leijten *et* al. [46]. Inside each microwell, a defined number of cells was brought into close contact with each other, without the possibility of cells moving from one microwell to another. This was important to accurately study the cell aggregation behaviour. In addition, due to the cylindrical shape and therefore flat bottom of the microwells, the influence of the gravity on the horizontal movement of cells (present in agarose inserts imprinted with concave microwells [27]) was negligible. Therefore, it does not interfere with cell aggregation kinetics. Bright-field microscopy enabled us to capture loosely connected cells as well as packed spheroids in a non-invasive manner. Using a 4X objective, ± 60 spheroids were observed in the field of view of a single frame. Due to slight movements of the inserts in the wells or imprecision of the stage, the spheroids moved in function of time. The proposed methodology was able to successfully track these spheroids over time, while spheroids that entered the field of view were correctly assigned to a separate matrix. However, spheroids that left the field of view and re-entered it later on were considered ‘new’ spheroids. In total, 48,000 spheroids could be cultured and simultaneously monitored in the 24 well plate. In that case, the minimum time interval between subsequent frames would be 15 minutes (i.e. with an exposure time of 50 ms, focus map and full well imaging).

### Quantitative assessment of the segmentation approach

The relative errors of the different features, computed to validate the automatic segmentation approach, are shown in Table 1 for each set. Especially for the area, minor axis length, major axis length and roundness, the algorithm obtained high accuracies (average error values ranging from 0.78–2.69%), while the perimeter and circularity were characterised by average error values between 2.49–4.50%. In Table 2, the sensitivities and precisions of the proposed segmentation approach were shown for each set. For the different sets, average sensitivities and precisions between 96.60–97.84% were obtained.

**Fig 3:**
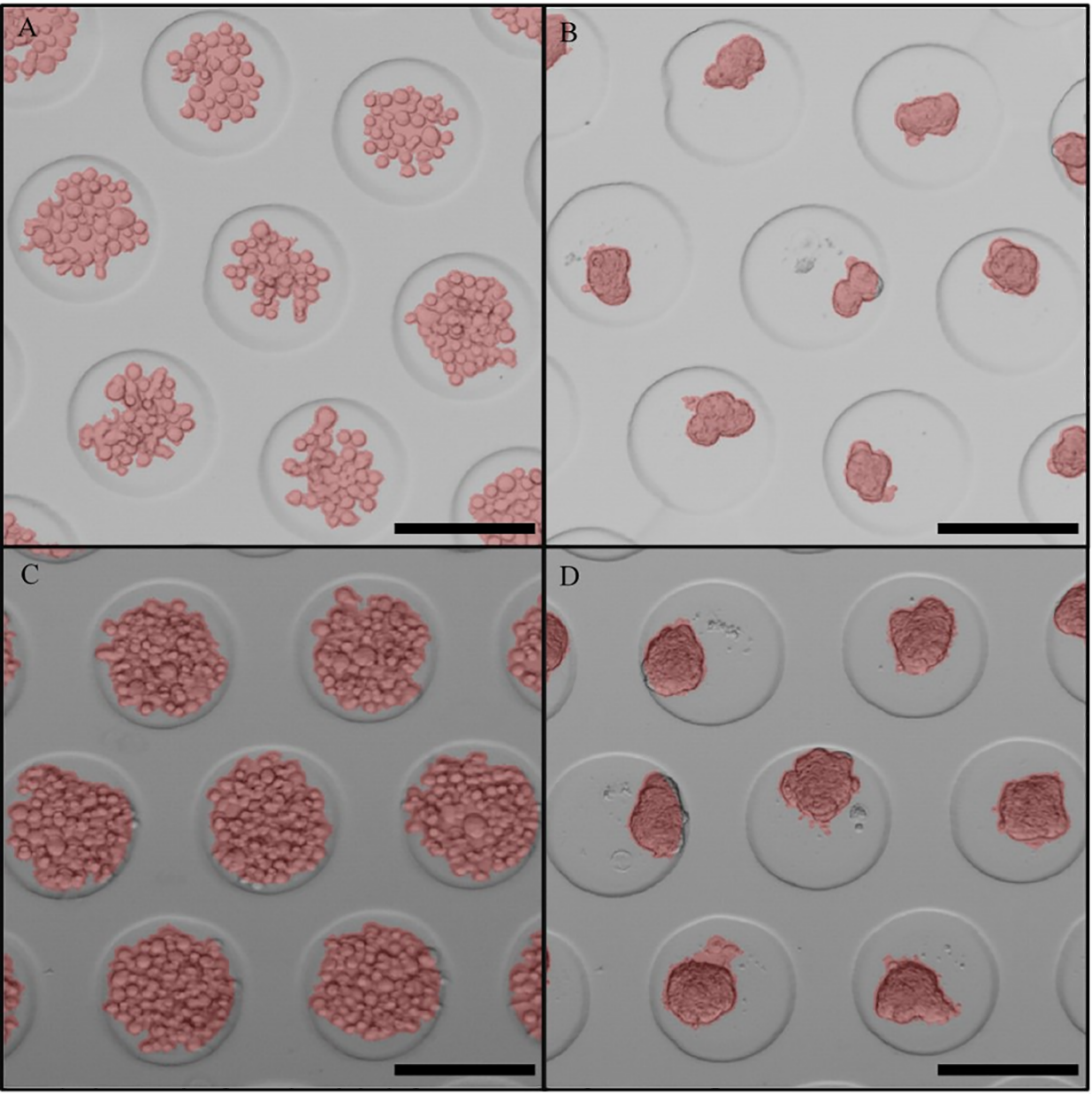
Qualitative assessment of the automatic segmentation approach. Semi-transparent, red-coloured masks of segmented spheroids overlaid on their corresponding raw image for: (A) Set 1; initial, small-sized spheroids. (B) Set 2; final, small-sized spheroids. (C) Set 3; initial, large-sized spheroids. (D) Set 4; final, large-sized spheroids. The black scale bars represent 200 μm.

For each set, seven spheroid masks (segmented by the algorithm) were overlaid on their corresponding raw image, as shown in Fig 3. In general, the masks overlapped accurately with the actual contours of the spheroids. It was observed that during the initial stage of the aggregation process, the individual cells were still visible (Fig 3A and 3C).

**Table 1:** Relative error values obtained for the extracted features.

	Set 1	Set 2	Set 3	Set 4	Set 1–4
Minor axis length	0.79 ± 0.73	1.09 ± 1.23	0.78 ± 0.62	1.73 ± 2.05	1.10 ± 1.33
Major axis length	1.04 ± 1.10	1.21 ± 1.59	1.79 ± 1.14	1.51 ± 1.44	1.38 ± 1.36
Roundness	1.22 ± 1.33	1.92 ± 2.04	2.00 ± 1.42	2.19 ± 2.27	1.83 ± 1.83
Area	1.65 ± 1.27	1.51 ± 1.76	1.44 ± 1.20	2.69 ± 2.41	1.82 ± 1.79
Perimeter	4.50 ± 3.75	2.66 ± 2.69	3.07 ± 1.86	3.86 ± 2.87	3.52 ± 2.94
Circularity	4.21 ± 3.50	2.49 ± 2.75	2.95 ± 1.83	3.34 ± 2.45	3.24 ± 2.76

For the different features, the relative errors were calculated between the segmentation results of operator–algorithm (accuracy). For each validation set, the results are represented as mean ± SD.

**Table 2:** Sensitivity (TPR) and precision (PPV) of the automatic segmentation approach.

	Set 1	Set 2	Set 3	Set 4	Set 1–4
TPR	0.9784 ± 0.0136	0.9721 ± 0.0228	0.9674 ± 0.0117	0.9667 ± 0.0303	0.9711 ± 0.0214
PPV	0.9719 ± 0.0122	0.9744 ± 0.0067	0.9773 ± 0.0056	0.9677 ± 0.0115	0.9728 ± 0.0100

Sensitivity and precision of the proposed methodology (operator–algorithm). For each validation set, the results are presented as mean ± SD.

### Time consumption

In Table 3, a comparison is made between the manual and automatic segmentation according to their time consumption. Due to differences in spheroid size and irregularity, a time interval of 35-60s was obtained for the manual segmentation (i.e. more time required for large, irregular spheroids). It took approximately 4 hours to manually segment the validation sets (in case no mistakes or breaks were considered). Extending this to the entire data would result in 1350–2300 hours of manual work, depending on the size and border irregularity of the spheroid. With the automated approach, one spheroid was analysed in approximately 0.65s. Therefore, the entire data was processed by the software tool in only 25 hours on a desktop computer.

**Table 3:** Comparative study between manual and automatic segmentation according to their time consumption.

	Manual segmentation	Proposed methodology
Single spheroid	35-60s	~ 0,65s
Validation set 1–4 (243 spheroids)	8505- 14580s	158s
All datasets (~139 000 spheroids)	1351-2317h	~25h

### Monitoring the morphological changes during cell aggregation on population and spheroid level

**Fig 4:**
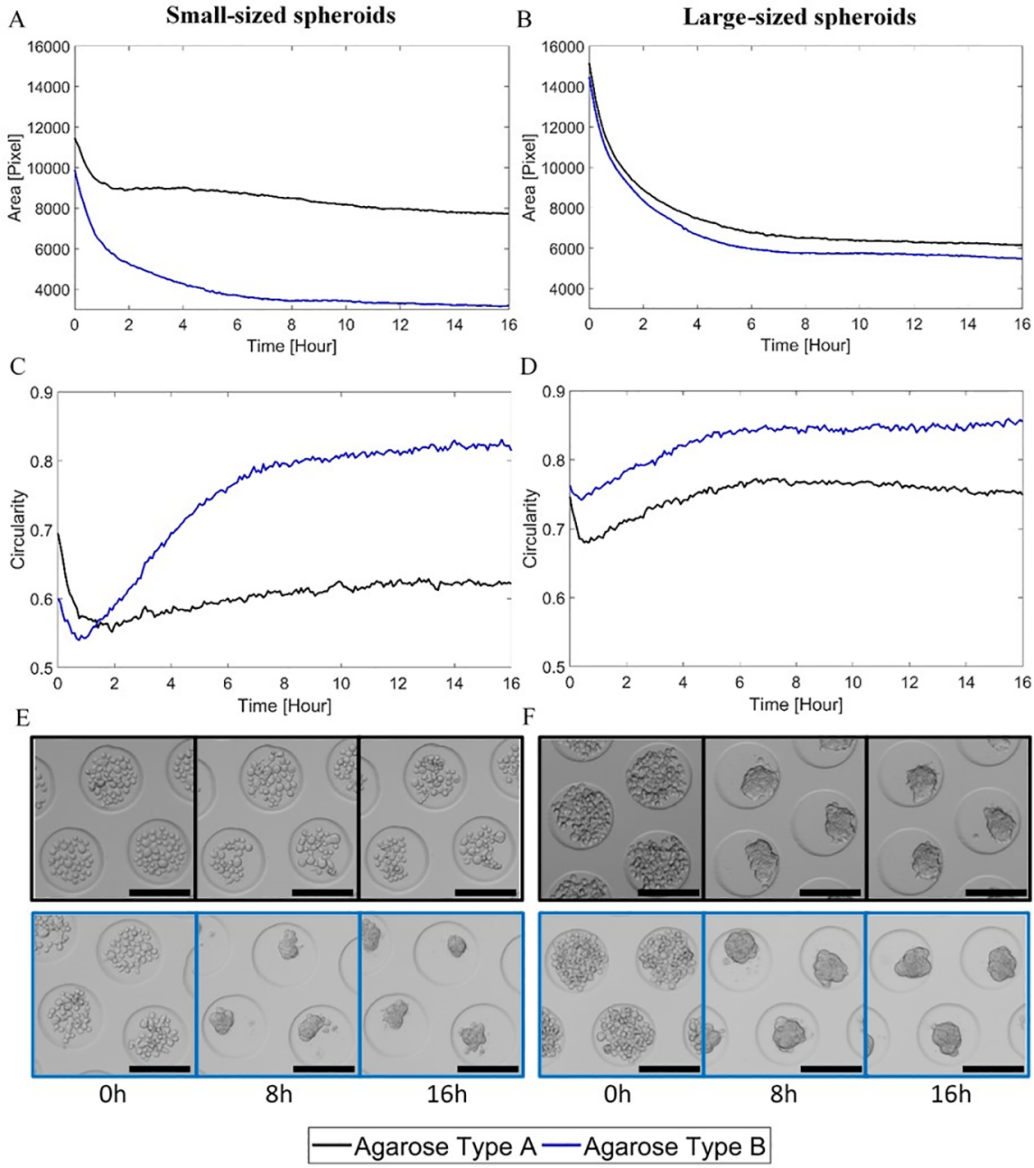
The average dynamic response of the spheroid area and circularity for each condition. (A), (B) Average response of the area for the two agarose types, respectively for the small- and large-sized spheroids. (C), (D) Average response of the circularity for the two agarose types, respectively for the small- and large-sized spheroids. (E), (F) Images of the cell aggregation process over time for the two agarose types, respectively for the small- and large-sized spheroids. The black scale bars represent 200 μm.

**Fig 5:**
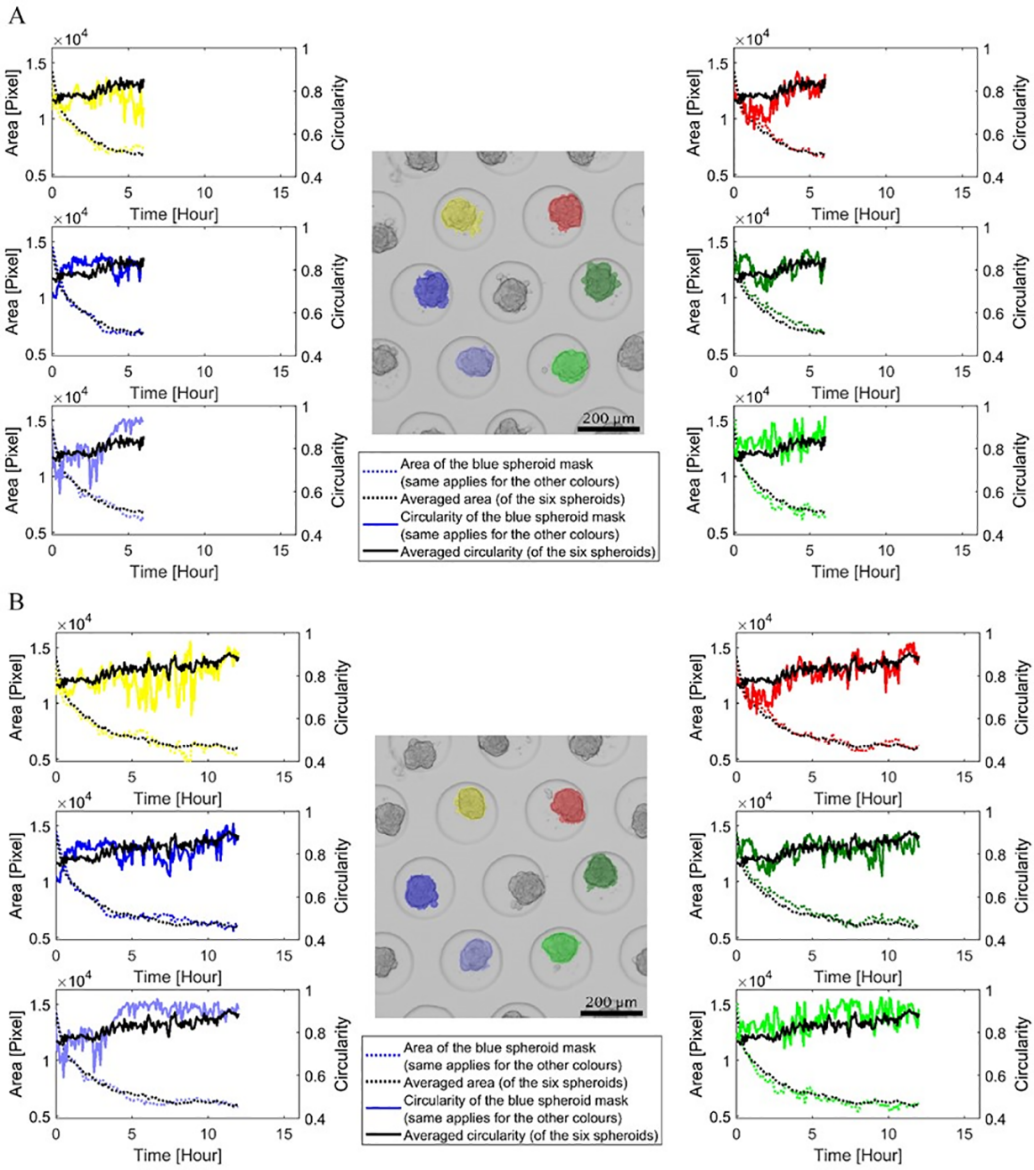
Illustration of the off-line monitoring process of individual spheroids in function of time. During cell aggregation, the dynamic response of the spheroid area and circularity was monitored. Here, six large-sized spheroids cultured on agarose type B were examined over time. Screenshots were taken after (A) 6 and (B) 12 hours. The black scale bars represent 200 μm.

Four different culture conditions were examined with approximately 180 spheroids per condition. Therefore, more than 700 individual spheroids were monitored over time using the developed system. In total, approximately 139 000 spheroid images (i.e. ~720 spheroids during 16 hours with time-lapse interval of 5 minutes) were automatically segmented and their corresponding features extracted. The responses of the spheroid area and circularity during cell aggregation were considered to be the most relevant and are therefore displayed in the following figures. In Fig 4, the average spheroid area and circularity were shown in function of time for each culture condition accompanied by their corresponding spheroid images at 0, 8 and 16h. For the small-sized spheroids, the decrease in area was much smaller for agarose type A than for type B, with a steady-state (i.e. variable of a process that does not change in time) area of respectively 7731 and 3207 pixels (Fig 4A). For the large-sized spheroids, the difference was less pronounced, with a steady-state area of respectively 6155 and 5474 pixels for agarose type A and B (Fig 4B). Another important feature was the circularity. In Fig 4C, the small-sized spheroids cultured on agarose type B exhibited a large increase in their average circularity (steady-state value at 0.815) compared to agarose type A (steady-state value at 0.622). The same observation applied to the large-sized spheroids (Fig 4D). Differences in the time-series data between conditions could also be related to differences observed in the time-lapse images after visual inspection. In Fig 5, the off-line monitoring of the cell aggregation process was illustrated on spheroid level (the complete monitoring process is shown in the S1 File). The dynamic response of the area and circularity was monitored for six large-sized spheroids, cultured on agarose type B. Each 5 minutes, a new image was acquired and processed, resulting in the continuous monitoring of the aggregation response. Comparing the individual aggregation responses with the average response, variability was observed. This was definitely the case for the circularity, exhibiting high frequency variations for the individual spheroids. These abrupt changes occurred mainly due to segmentation errors or the fact that cells and cell clusters attached to/detached from or integrated into the main spheroid, as can be observed in the S1 File.

## Discussion

Quantification of the cell aggregation kinetics under variable process conditions can provide useful information and could give rise to new insights in spheroid biology. Additionally, enabling technologies such as the one developed in this study, are needed to follow up the formation of these microspheroids during large-scale manufacturing. Keeping this in mind, bright-field microscopy without additional sample preparation or staining was applied, conducting a non-invasive operation. Among the different culture techniques (e.g. suspension cultures, hanging drop method, rotating wall vessel) that enable the formation of 3D spheroids [16], the agarose microwell format was selected. It is a scalable technique that provides uniformly sized spheroids and allows real time monitoring of the cell aggregation process [43]. In the 24 well plate, approximately 48,000 spheroids can be produced and simultaneously monitored, achieving a much higher throughput than systems using e.g. well plates with a single spheroid per well [12][34][35][36]. In addition, there is also a large improvement in terms of the minimum time-lapse interval. The system described in Vinci *et al*. [35] required 8 minutes for scanning a 96 well plate (one compacted spheroid in each well), while our system can capture up to 48,000 spheroids within an interval of 15 minutes. Combining these characteristics with a software tool to automatically analyse the captured data, a platform for high-throughput monitoring of spheroid formation was established. It is important to notice that the achieved throughput is a direct result of the microwell format in combination with the imprinted microwells of 200 micrometre diameter, which in turn puts a restriction on the size of the spheroids. In our case, spheroids up to 150 micrometre can be obtained. These sizes are relevant in the field of tissue engineering, since they give rise to a viable microtissue [46]. For microspheroids with a diameter exceeding 200 micrometre, more inner cell necrosis is observed due to impaired nutrient and oxygen diffusion [53][54].

The segmentation approach was validated on four sets of ± 60 spheroids, including loosely connected cells present at the onset of spheroid formation and readily formed spheroids, for both spheroid sizes investigated. By considering two different spheroid sizes, the performance of the algorithm could be evaluated for a microwell surface partly covered (i.e. small-sized spheroids) or completely covered (i.e. large-sized spheroids) with cells. The validation results for the different sets confirm that our system can be applied to accurately monitor the cell aggregation process. In general, the relative errors on the perimeter and circularity were higher compared to the other features. This can be attributed to the higher sensitivity of these features to segmentation errors. In Piccinini *et al*. [34], a semi-automated approach for spheroid analysis was described and validated on three sets of 50 mature spheroids. At first sight, the relative errors, sensitivities and precisions achieved by Piccinini *et al*. compared closely to the values obtained in our study. In their study, slightly higher average sensitivities and precisions (i.e. 96.93–99.90% vs 96.60–97.84%) were obtained. However, in our approach a much higher throughput (>139 000 spheroids) was established with a minimum of manual workload. Moreover, their analysis was restricted to relatively mature spheroids, while the initial stages of cell aggregation (i.e. where single cells are still visible) exhibit lower contrast and are therefore more challenging to segment accurately. Subsequently, a qualitative assessment was performed to gain more insight regarding the automatic segmentation process and the introduced errors. At the initial stage, ‘holes’ (i.e. regions lacking cells) often appeared inside the spheroid, which were filled up prior to feature extraction. Small notches that occurred in the spheroid (Fig 3A and 3C) were also considered to contribute to the incompactness of the spheroid and were therefore closed and filled up (see Image analysis subsection for more information). The introduction of the observed errors was partly due to the occurrence of low to non-contrast regions at the boundaries of the spheroid (Fig 3A), imposing a challenge for segmentation algorithms. In addition, it was also challenging to accurately remove the microwell border due to its irregular thickness, caused by shade artefacts (introduced by optical microscopy) and out of focus regions (due to thickness variations along the insert). In case the spheroid approached or overlapped with the microwell border, it was possible that a part of the segmented spheroid mask was cut off (Fig 3C and 3D) or a part of the microwell border was still connected to the spheroid.

Manual segmentation is a time-consuming (costly) and monotonous process, thereby preventing the analysis of large image datasets in a robust fashion. For our datasets, manual segmentation would take 1500–2300 hours, while the developed tool only required ~25 hours of processing. Therefore, the algorithm performs on average 73 times faster than a human operator. In addition, manual segmentation is also affected by segmentation errors and subjective interpretation of images. In our case, the uncertainty of the operator for closing notches in the image of the spheroid and connecting smaller cell regions (in proximity of the main spheroid) to the main spheroid (Fig 3D), could result in an additional error. Using quantitative software tools, the consistency of results can be increased. In our methodology, the segmentation parameters are fixed (i.e. the gradient threshold) or automatically-defined (i.e. the intensity threshold) to eliminate intra- and interoperator variabilities and therefore yield more consistent results. A minor drawback of these fixed parameter settings is a decrease in adaptability of the algorithm to certain edge-case situations. In order to further minimise the interference of a human operator, the proposed system was equipped with an automated scanning stage. This in contrast to the method described in Piccinini *et al*. [34], considered a labour intensive approach due to the manual acquisition of images containing a single spheroid.

As a proof of concept, the cell aggregation process was monitored for four different culture conditions. Surprising was the average response of the area of the small-sized spheroids cultured on agarose type A. In contrast to the other conditions, the aggregation process was interrupted for this culture condition resulting in loosely connected cells throughout the experiment. In addition, for both spheroid sizes, the average circularity of spheroids cultured on agarose type A seemed drastically lower compared to type B. This could provide information on the stability of the spheroid. However, the experiment was solely conducted to support the proposed methodology, therefore no conclusions will be drawn from the obtained results. This study of the cell aggregation dynamics can be generalised to other applications involving different process conditions [55] or cell types [56] of interest.

One of the most interesting aspects of the developed system is monitoring the formation of individual spheroids over time, directly relating to the high-throughput of the approach. Due to the heterogeneity present in cell-based experiments, single spheroid monitoring systems are considered important. To the authors’ knowledge, it was the first time that cell aggregation dynamics were automatically monitored for individual spheroids. In total, the aggregation behaviour of more than 700 individual spheroids was monitored in this study. As previously mentioned, the system has the ability to continuously monitor up to approximately 48,000 microspheroids. For this reason, it can be useful as a high-throughput screening platform. In Wong *et al*. [36], an *in vitro* drug screening was performed using a 24 well plate, containing a single spheroid per well. In total, five different drug dosages were examined in parallel over time with four replicates for each condition. In contrast to Wong *et al*., our system is able to screen up to 24 different conditions (i.e. 24 wells) in parallel at a much higher sampling frequency, with a maximum of 2,000 replicates per condition. It is important to notice that in the study of Wong *et al*., spheroids of approximately 300–400 micrometre in diameter were used, while the microwell diameter of our inserts is only 200 micrometre. However, it has been shown, for example in Patra *et al*. [57], that these smaller spheroids can also be useful for drug screening applications. In addition, we also believe that our methodology could be applied to larger microwells sizes (e.g. up to 800 micrometre), which in turn enables the production of larger spheroids. This would still result in a drastic increase in throughput compared to conventional systems.

Microspheroids are of great interest in bottom-up tissue engineering, where they can be used as building units to produce large tissue constructs. A first step in this process is to achieve a better understanding and characterisation of spheroid formation. In general, this process is still characterised based on static measurements, lacking essential information on the kinetics of spheroid formation [22][56]. Therefore, it is essential to monitor the aggregation process over time. Using the developed methodology, more insight into the kinetics of cell aggregation and the formation of 3D tissues can be obtained. Furthermore, the system could contribute towards the implementation of monitoring and control strategies in the biomanufacturing of spheroids (concept illustrated in Fig 1). For example in differentiation protocols, spheroid size has important implications on the differentiation potential of stem cells [30][43]. Monitoring and eventually controlling spheroid formation is therefore a prerequisite to ensure a biologically relevant outcome.

The time-varying morphological features, automatically extracted for each spheroid, can be further processed using data-based modelling techniques [58]. In this way, parameters relating to the aggregation kinetics can be obtained from the data. Another use for the obtained features could be the validation of cellular aggregation results obtained through *in silico* simulation [59].

## Conclusion

A methodology was proposed for the automated, non-invasive monitoring of the morphological changes during cell aggregation. As a proof of concept, the aggregation behaviour of single cells was monitored for different process conditions (i.e. spheroid size and agarose type). More than 700 individual spheroids were successfully tracked through the imaging datasets. Therefore, the achieved throughput significantly outperformed other systems focusing on compact spheroids, which were often restricted to 96 spheroids. In order to evaluate the performance of our approach, a quantitative validation was performed. For the different validation sets, the algorithm achieved average sensitivities and precisions ranging from 96.60–97.84% and average relative errors between 0.78–4.50% for the morphological features under consideration. These results demonstrate the system’s potential to monitor the formation of individual spheroids in biomedical research and manufacturing protocols, thereby reducing the high work-load associated with manual analysis of large scale, spheroid-based assays. In addition, it can open up the way for the implementation of new prediction and control strategies, thereby significantly improving the biofabrication of microspheroids.

## Supporting information

S1 Table Description of the features extracted from the spheroids. For a more detailed explanation of the different features, have a look at the ‘regionprops’ documentation on the MathWorks website.
(DOCX)

S1 File Video of the off-line monitoring of the aggregation process. (AVI)
