# Supplementary material for: High-throughput image-based monitoring of cell aggregation and microspheroid formation
Source: PLoS One. 2018 Jun 28;13(6):e0199092. doi: 10.1371/journal.pone.0199092 (PMC6023212; doi:10.1371/journal.pone.0199092)
Supplement: S1 Table — For a more detailed explanation of the different features, have a look at the ‘regionprops’ documentation on the MathWorks website. (DOCX) [file pone.0199092.s001.docx]

| Extracted features | Description [Unit] | Visual representation |
| --- | --- | --- |
| Minor axis length | The length of the minor axis of the ellipse that has the same normalized second central moments as the region [Pixel]. | 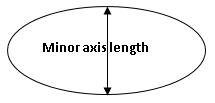 |
| Major axis length | The length of the major axis of the ellipse that has the same normalized second central moments as the region [Pixel]. | 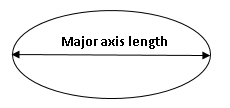 |
| Roundness | The roundness was computed according to the following formula:  $Roundness= \frac{Minor axis length}{Major axis length}$  The value ranges from 1 (circle) to approximately 0 (infinite line segment). |  |
| Area | The number of pixels in the region [Pixel]. |  |
| Perimeter | The length of the outline of the region [Pixel]. |  |
| Circularity | The circularity was computed according to the formula proposed in Kelm *et al.*^58^:  $Circularity= \frac{\pi*\sqrt{\frac{4*Area}{\pi}}}{Perimeter}$  The value ranges from 1 (circle) to approximately 0. The circularity can be interpreted as the deviation with respect to a circle. |  |
| Additional features | **Description [Unit]** | **Visual representation** |
| X Centroid | Horizontal coordinate (x-coordinate) of the region its center of mass. |  |
| Y Centroid | Vertical coordinate (y-coordinate) of the region its center of mass. |  |
| Orientation | The angle ($\theta$) between the horizontal axis and the major axis of the ellipse that has the same normalized second central moments as the region. The value ranges from -90° to 90° [Degree]. | 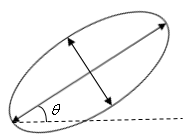 |
| Mean intensity | The mean of all the intensity values that are part of the region. |  |
